# Single-ion adsorption and switching in carbon nanotubes

**DOI:** 10.1038/ncomms10475

**Published:** 2016-01-25

**Authors:** Adam W. Bushmaker, Vanessa Oklejas, Don Walker, Alan R. Hopkins, Jihan Chen, Stephen B. Cronin

**Affiliations:** 1 Physical Sciences Laboratories, The Aerospace Corporation, 355 S. Douglas Street, El Segundo, California 90245, USA; 2 Department of Electrical Engineering, The University of Southern California, 3601 Watt Way, Los Angeles, California 90089, USA

## Abstract

Single-ion detection has, for many years, been the domain of large devices such as the Geiger counter, and studies on interactions of ionized gasses with materials have been limited to large systems. To date, there have been no reports on single gaseous ion interaction with microelectronic devices, and single neutral atom detection techniques have shown only small, barely detectable responses. Here we report the observation of single gaseous ion adsorption on individual carbon nanotubes (CNTs), which, because of the severely restricted one-dimensional current path, experience discrete, quantized resistance increases of over two orders of magnitude. Only positive ions cause changes, by the mechanism of ion potential-induced carrier depletion, which is supported by density functional and Landauer transport theory. Our observations reveal a new single-ion/CNT heterostructure with novel electronic properties, and demonstrate that as electronics are ultimately scaled towards the one-dimensional limit, atomic-scale effects become increasingly important.

## Abstract (web summary)

Single ion detection is typically performed with large devices rather than microelectronic devices. Here, the authors report the electrical detection of gaseous ions on single isolated carbon nanotubes, with a mechanism proposed based on ion-induced charge depletion in the nanostructures.

## Graphical abstract

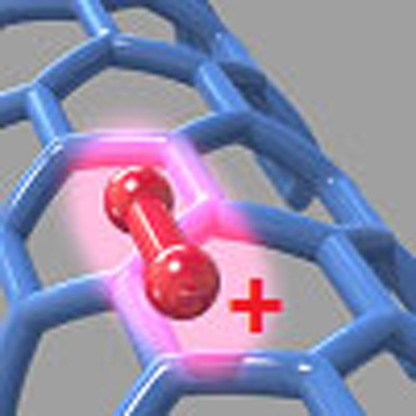

Electronic conduction in one-dimensional (1D) channels has been the focus of many research efforts over the past 15 years, and during that time carbon nanotubes (CNTs) have often served as the prototypical system for studying 1D conduction. The reason for the strong interest is both because of the interesting fundamental physics that occur in such systems^1^,^2^,^3^ and because of the enhanced functional performance that has been predicted to occur in CNT-based field effect transistors (FETs) such as high linearity and high operation frequency^4^,^5^. In addition, CNT devices have been developed for new applications using the unique properties of this new material, such as sensors^6^, radio frequency (RF) nanoelectromechanical (NEM) devices^7^ and flexible electronics and displays^8^. CNT FETs are now being considered as a promising new technology for next-generation micro- and nano-electronic devices and circuitry.

Owing to their small physical size, the conductivity of CNTs and other molecular systems is highly susceptible to changes in charge states of defects nearby or in contact with the material^9^, or even by direct chemical activity on the surface of the CNT^10^,^11^. Similar switching behaviour has also been observed in molecular electronic systems^12^, and also as random telegraph noise in scaled deep-submicron silicon devices^13^. As dimensions in nanoelectronic devices are scaled further and further towards the single-atom, 1D channel limit, effects such as those listed above will become increasingly important, even to the point where they dominate the device operation. Single neutral atom detection techniques have shown only small, barely detectable responses^14^,^15^,^16^,^17^, and there is relatively little information in the literature on ion adsorption on the surfaces of solids from gas. This is despite the various industrial techniques that make use of ions from corona discharge for surface treatment, such as a surface treatment of polymers to improve adhesion and other surface properties^18^, and surface charging of insulators as a contactless electrode for both processing and characterization in the semiconductor microelectronics industry^19^,^20^. Liquid-phase ion–surface interaction is also an important topic, for applications such as ionic gating of graphene^21^.

In this study, we report the effect of single gaseous ion adsorption on the conductance of isolated, suspended, single-walled CNT FETs, which were electrically characterized *in situ* during exposure to gaseous ions created by ionizing radiation and high-voltage corona discharge. Positive ions are found to increase the resistance of the CNT by several orders of magnitude, while negative ions have no effect on electrical conduction. Multiple simultaneous ion adsorption events are detected, leading to quantized increase in total device resistance. The gate voltage dependence of electrical conduction during ion adsorption is measured and characterized, and *in situ* electrical heating of the suspended CNTs is used to drive off adsorbed ions. These experimental results are modelled computationally using density functional theory (DFT) and Landauer electrical transport theory.

## Results

### Electrical current during exposure to gaseous ions

**Figure 1:**
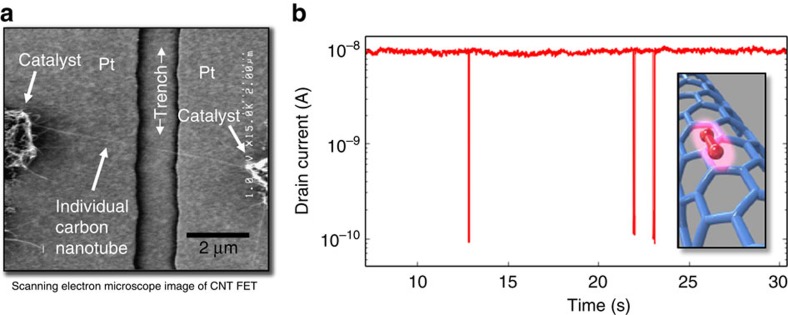
Device layout and switching transients caused by single-ion adsorption. (**a**) Scanning electron microscope image of CNT FET device and (**b**) plot of drain current versus time showing switching transients observed during ionized gas exposure. The inset shows a cartoon image of a gas molecule adsorbed on the surface of a carbon nanotube.

Figure 1a shows a scanning electron image of one of the CNT FET devices used in this study. The CNT is suspended over the trench, making electrical contact on either side, with a gate electrode in the bottom of the trench. Figure 1b shows the drain current plotted versus time for the CNT, showing large decreases in drain current observed during exposure to ionized nitrogen gas. The observed transients are characterized by sudden, discrete reductions in current, which had durations ranging from milliseconds to minutes, followed by an equally sudden recovery back to the pristine state. The transient events were observed during exposure to ionized air, Ar, N_2_, He and O_2_, and ceased occurring when the surrounding gas was removed using a vacuum, or when the source of ionization was removed.

### Multiple ion interactions

**Figure 2:**
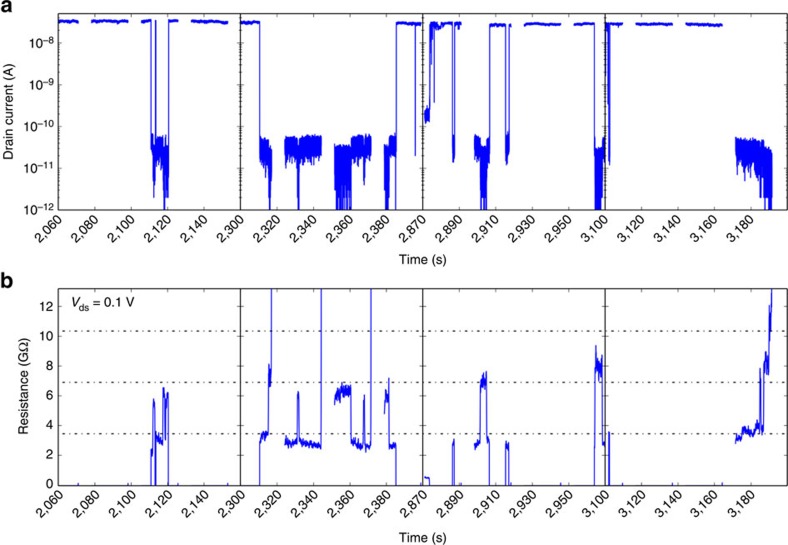
Drain current and calculated resistance plotted versus time. (**a**) The drain current shows numerous sharp reductions in magnitude, and (**b**) calculated resistance shows quantized equally spaced resistance states. The resistance was calculated using Ohms law with the drain-source voltage of 0.1 V. To reduce noise, the displayed resistance is a running average of 50 data points.

In Fig. 2a,b, the device current and resistance during exposure to higher gas ionization rates are plotted, showing multiple, simultaneous switching events, each adding a quantized resistance of *R*_ion_ to the total device resistance. The current was measured every 4 ms using a two-point method, and the resistance was calculated using Ohms law with the drain-source voltage of 0.1 V. To reduce noise, the displayed resistance is a running average of 50 data points. After collection of each data set, there is a dead period during which data are transferred to the computer. Equally spaced dashed lines separated by 3.45 GΩ were added to Fig. 2b as a guide to the eye, highlighting the quantized steps in resistance.

**Figure 3:**
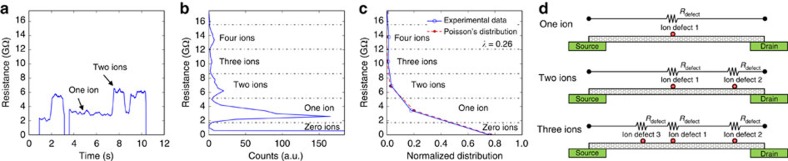
Statistical analysis of multi-level switching and Poissonian model for ion adsorption. (**a**) Resistance plotted versus time for an exemplary data set of 5,000 data points showing multi-level switching. (**b**) Resistance measurement histogram for an entire ion exposure period including 50 total data sets similar to those shown in **a**. (**c**) Normalized distribution of data (solid blue line with open circles) in binned resistance states corresponding to observed levels in **b**, plotted along with a fit Poissonian distribution (red dashed line, solid circles), and (**d**) Poissonian model for multiple, independent, simultaneously adsorbed ions. The ion defect resistance for this data set was found to be 3.45 GΩ.

Figure 3a shows a close up of exemplary data from the first frame in Fig. 2b. To analyse the statistics of the multi-level switching data, a histogram of resistance levels was calculated for the entire period of exposure to ions, with bin widths of 400 MΩ. This analysis encompassed 50 data sets such as those shown in Fig. 3a, or 1,000 s of time series data. Grouping can clearly be observed in the resistance data, with one dominant peak at the axis (actual resistance is ∼0.003 GΩ), two prominent peaks at ∼3 and 6 GΩ and two smaller peaks at 10 and 14 GΩ, corresponding to the quantized steps in resistance observed in Fig. 2. The average peak spacing for all peaks observed was found to be *R*_ion_=3.45 GΩ. These five peaks are attributed to the pristine CNT resistance, and the resistance of the CNT with one, two, three and four ions, respectively, adsorbed to the CNT surface at different locations. If the arrival of individual ions on the CNT surface is uncorrelated, as one would expect, then the probability of observing a given number of adsorbed ions in any given measurement should form a Poisson's distribution. To test this hypothesis, the measured resistances were tabulated into bins separated by *R*_ion_ (dashed lines denote bin edges), the distribution normalized and the result plotted in Fig. 3c (solid blue line with open circles). A Poisson's distribution was also fit to the data, given by


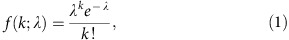


where *f* is the probability of occupation, *k* is the number adsorbed ions and *λ* is the expected value for individual ion adsorption, and also the only fitting parameter. The resulting Poisson's distribution was plotted in Fig. 3c (dashed red line with solid circles), along with the fit value *λ*=0.26. As can be seen, the data fit a Poisson's distribution well, with the exception of the anomalous peak of four ions, which were observed as a single long switching event in one data set. To evaluate the goodness of fit, the *χ*^2^-value was calculated for a fit including all resistances shown (*k*=0 through 5) and was found to be 1.87. However, when we ignore the outlier data set mentioned above by only considering data below 12 GΩ (*k*=0 through 3), the calculated value for *χ*^2^ is only 0.028, indicating a better fit, and that these data points are well described by a Poisson's distribution. A cartoon model illustrating multiple ion defects along the length of the CNT is shown in Fig. 3d, and explains this observed behaviour. Individual ions make their way through diffusive transport to the surface of the CNT and adsorb independently, each adding a resistance of several GΩ in series to the total resistance of the CNT.

### Switching event duration

**Figure 4:**
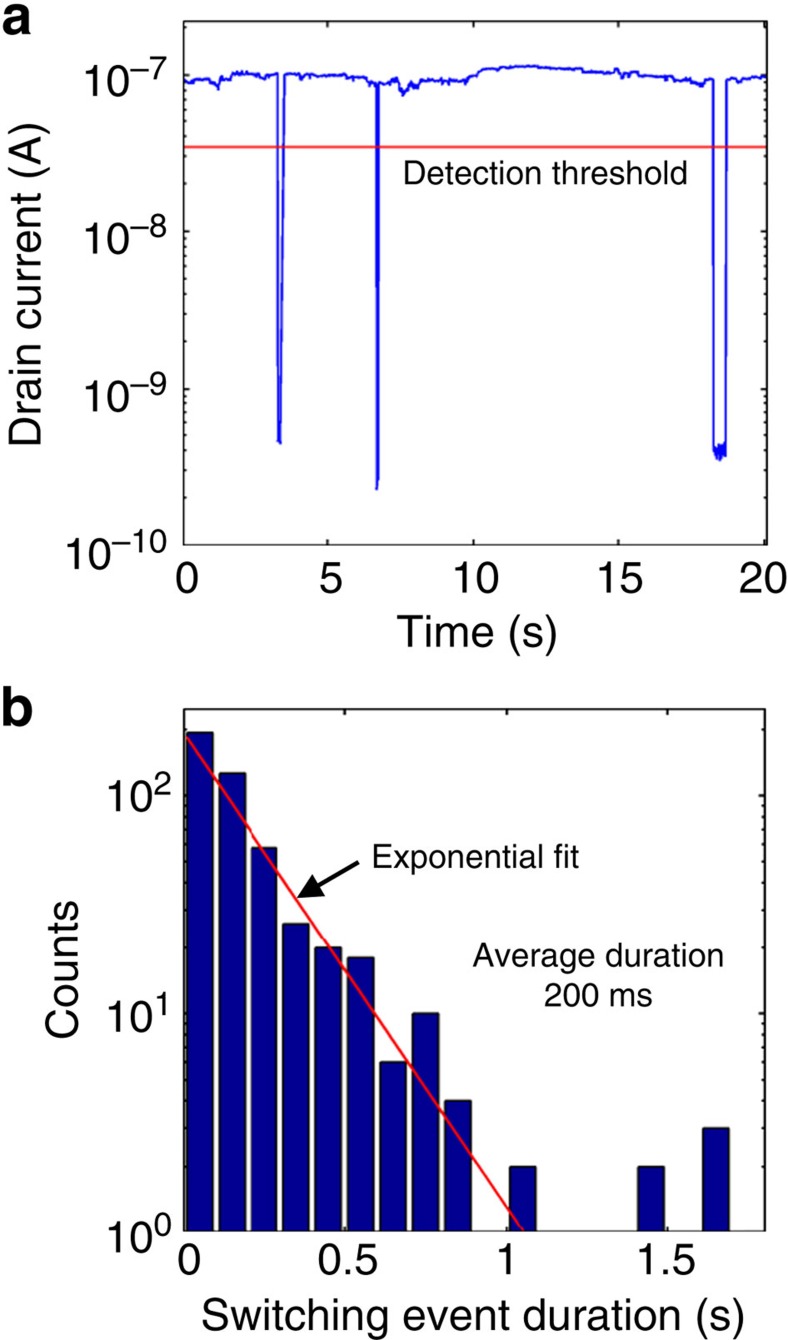
Statistical analysis of ion residency time. (**a**) Drain current plotted versus time during gaseous ion exposure, with the threshold for event detection denoted with a red line, and (**b**) histogram plotting duration of a large number of switching events, with exponential fit. The average switching event duration for this experiment was found to be 200 ms.

To investigate the statistics of desorption time, the duration of large number of switching events was measured during exposure to gaseous ions at a lower ionization rate, and plotted in Fig. 4. The drain current is plotted versus time for an exemplary data set in Fig. 4a. For lower ionization rates and shorter adsorbed ion residency times, multiple steps are not observed because of the decreased probability for multiple simultaneous interactions. A total of 475 adsorption events were recorded over a time period of 3 h and 10 min. The events were automatically characterized by a computer algorithm, which calculated the total time the current for each event spent below a detection threshold (noted in Fig. 4a). The event duration varied from event to event, and the distribution of event durations calculated for this data set is shown in Fig. 4b. The number of observations for a given event duration decays exponentially, with an average duration of 200 ms. Similar switching behaviour has been observed before in CNT FETs^9^,^22^, as random telegraph signal (RTS). RTS behaviour is somewhat commonly observed in both CNT FET and conventional scaled metal oxide semiconductor MOSFET devices, and is attributed to charge fluctuations in defects at the semiconductor/oxide interface. The associated capture and emission times for typical RTS systems take an exponential probability distribution (as do the adsorbed ion residency durations here), and are determined by the temperature and state energy^13^. In this context, the average ion residency duration can be interpreted as the emission time for a random telegraph-like system, where the emission time corresponds to the gaseous ion desorbing from the CNT surface.

### Temperature effects

**Figure 5:**
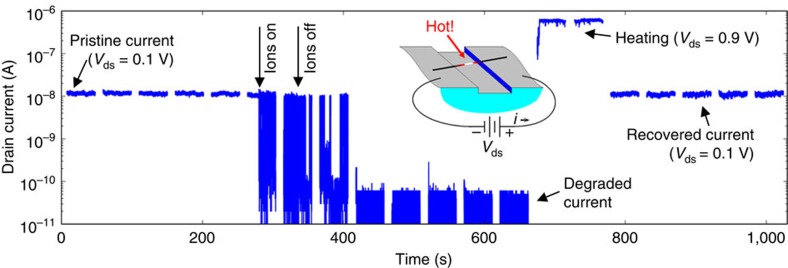
Ion adsorption and *in situ* thermally induced desorption. Drain current data plotted versus time before, during and after ion exposure and local electrical heating. After ion exposure, the CNT remains in a high resistance state until application of high-voltage bias, which heats the suspended CNT. The estimated temperature increase during the electrical heating phase is 45 °C.

To investigate the effect of temperature on the adsorbed ion residency, electrical current was passed through a CNT FET device, increasing the temperature of the suspended CNT *in situ* through Joule heating. Figure 5 shows data from this experiment, in which the device was exposed to a high flux of argon ions generated by corona discharge. After ion exposure, the device resistance increased substantially, remaining in its high resistance state for several minutes. After this period of persistent degradation, a larger drain-source voltage of 0.9 V was applied to the device for a period of 80 s, inducing 600 nA of electrical current to flow through the device, heating the CNT in the process. After this treatment, the device resistance was restored to its pristine state. This switching and recovery cycle was repeated four times. Eventually, the device did not recover, possibly because of irreversible chemical or structural changes in the device during prolonged exposure to elevated temperatures. From previous measurements on similar CNT devices using Raman spectroscopy as an *in situ* temperature probe^23^,^24^, the electrical power dissipated in the device during the heating steps shown in Fig. 5 (540 nW) is estimated to increase the temperature of the CNT by ∼45 °C above ambient room temperature. Under such bias conditions, the majority of electrical power dissipation occurs in the CNT itself, as opposed to the contacts because of strong optical phonon scattering^25^. It is possible that ion desorption is assisted by nonequilibrium electron and phonon populations in the CNT, which have been shown to occur in CNT devices at high-voltage biases^26^.

### Ion polarity effects

To determine the polarity of the ionic species interacting with the CNT, an ionization drift chamber was constructed, and electric fields were applied across the chamber containing the CNT and the surrounding ionized gas. This allowed us to drive either positive or negative ions towards the CNT, depending on the polarity of the electric field. Only positive ions were found to cause the transient switching events to occur, while electrons and negative ions did not (see Supplementary Fig. 1). The electric field applied was 17.3 V cm^−1^. Ions were not accelerated significantly above room temperature thermal energy, and the electric field is not expected to influence adsorption. Further details on ion polarity measurements are described in Supplementary Note 1.

### Contact effects

All data collected in the near-threshold regime of device operation (gate voltage data near *V*_G_=0 V and all time series data) displayed a pristine device resistance of several MΩ. The contact resistance in CNT FET devices with Pt Schottky contacts (such as those used in this study) is in the range of several tens to hundreds of kΩ (refs 27, 28), which is relatively small compared with the pristine channel resistance (to say nothing of the several GΩ device resistance during exposure to ions). Thus, the contacts are not expected to play a major role in the transport phenomenon observed here. It is possible that the ions interact with the contacts, increasing the contact resistance of the CNT devices. This is unlikely, however, because of the fact that up to four resistance quanta are observed, and there are only two contact resistances to be modified (source and drain). In addition, it is unlikely that a single ion would have a strong effect on the Pt metal in the contact because of charge screening from the large free electron density in the bulk metal. Finally, the observed change in subthreshold swing requires some interaction with the gate. Ions on the surface of the metal contacts would be screened from the gate by the large free electron density in the metal nearby.

### DFT modelling

**Figure 6:**
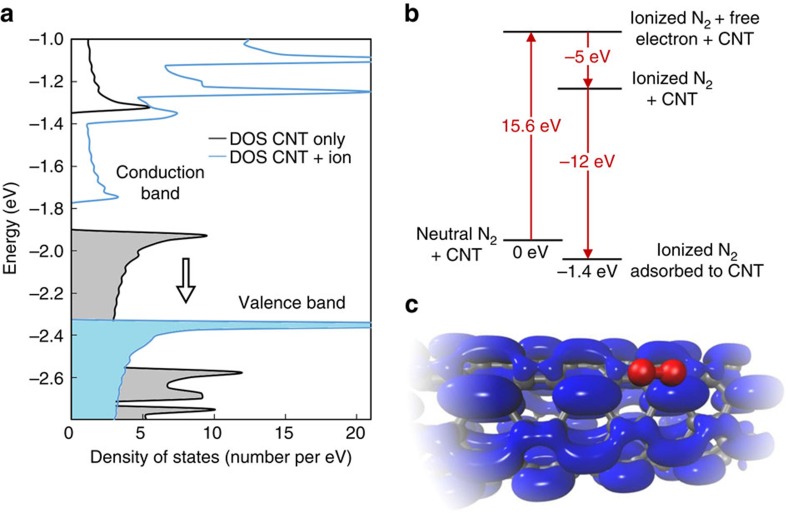
Density functional theory results. (**a**) Calculated density of states with (cyan) and without (black) adsorbed ion plotted versus energy, (**b**) ionization/adsorption energy diagram for N_2_^+^ adsorbed to the surface of an (8,0) CNT and (**c**) electron density plot for the gamma point conduction band wavefunction on the CNT with adsorbed N_2_^+^ ion. The equilibrium adsorbed ion–CNT separation was found to be ∼3 Å, with significant charge transfer of 0.8 electrons to the adsorbed ion. The large binding energies found for ion adsorption on CNTs indicate quasi-stable ion residency, which explains the long (milliseconds to minutes) observed lifetimes.

To model the effects of adsorbed ionic species on the electrical properties of the CNT, DFT modelling was performed using a 1.4-nm-long section of an (8,0) semiconducting CNT with and without adsorbed N_2_^+^. First, calculations were performed to find the equilibrium CNT–N_2_^+^ configuration, which gave an equilibrium ion–surface separation of 3.0 Å (ion distance to the CNT axis was 6 Å). Results indicate that the binding energy for N_2_^+^ on the surface of the CNT is −11.95±0.02 eV, which is considerably more favourable than the binding energy for neutral N_2_ (−0.16±0.02 eV). Lowdin charge analysis shows that there is also significant charge transfer to the ion of 0.8 electrons from the CNT to the ion. The densities of states for the pristine CNT and CNT with adsorbed N_2_^+^ are plotted in Fig. 6a, in which the CNT–N_2_^+^ band states are shifted down by ∼450 meV. It is hypothesized that, despite the presence of free electrons on the CNT and significant charge transfer to the adsorbed ion, the ion does not recombine with an electron to form a free neutral molecule because the total system energy is lower for the adsorbed ion state, as outlined in Fig. 6b. For more information on DFT calculations, see Supplementary Note 2 and Supplementary Fig. 2.

### Gate voltage dependence and electrical transport modelling

**Figure 7:**
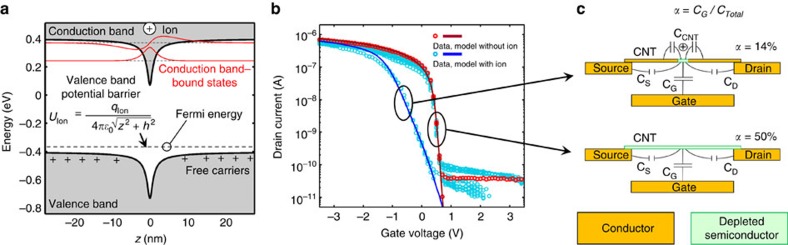
Ion potential and gate voltage dependence. (**a**) CNT potential plotted versus distance along the CNT axis with adsorbed ion. (**b**) Drain current plotted versus gate voltage showing experimental data from CNT in both pristine (red circles) and defected (cyan circles) states, along with corresponding results from a Landauer transport model (dark red and dark cyan lines) of the CNT with the adsorbed ion potential shown in **a**. (**c**) Lumped capacitance model for pristine and ion-adsorbed subthreshold CNT FETs. The ion charge *q*_Ion_ and separation distance *h* used to calculate the results plotted in **a**,**b** were 0.18 elementary charges and 7 Å, respectively, in concordance with DFT results.

Data showing the gate voltage dependence of the CNT FET drain current in both the pristine state (without ions) and transient switched state (during ionized gas exposure) are plotted in Fig. 7b. Data taken while the CNT was in the pristine state are represented as red circles; data taken while the CNT was exposed to ions are represented by cyan circles; and the dark cyan and dark red lines represent a theoretical transport model, discussed below. The pristine CNT FET shows *p*-type behaviour, with the device being ON for negative gate voltages and an ON/OFF ratio of ∼1 × 10^4^. The devices are not strained significantly by the application of gate voltage, as determined by Raman spectroscopy^29^. The subthreshold swing of the drain current in the pristine state is ∼110 mV per decade. The gate voltage dependence of the drain current from the defected CNT state is similar to that of the pristine state, although the threshold voltage is shifted more negative, and the subthreshold swing is increased substantially to 400 mV per decade. Both data sets are fit by a theoretical transport model, as outlined below. The negative threshold voltage shift is caused by the presence of positive charge near the 1D channel, and the increase in subthreshold swing is characteristic of the lowered effective gate efficiency for the defect. The subthreshold swing for a FET, which is the reciprocal value of the subthreshold slope, is described by


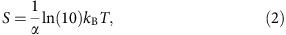


where *α*=*C*_G_/*C*_Total_ is the gate efficiency, with gate capacitance *C*_G_ and total capacitance *C*_Total_.

To model the effect of the added ion potential on device electrical characteristics with greater fidelity, numerical calculations were performed to solve the discretized quantum mechanical Hamiltonian for the electron eigenstates and quantum mechanical carrier transmission coefficients^30^ for the new system consisting of CNT with adsorbed ion potential *U*_Ion_. On the basis of these results, a Landauer transport model was used to calculate the gate voltage dependence of the drain current in a CNT with an adsorbed ion, where the potential on the CNT is calculated by solving Poisson's equation^31^:


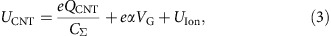


where *Q*_CNT_ is the charge on the CNT, *V*_G_ is the gate voltage and *U*_Ion_ is the potential associated with the adsorbed ion, which is given by


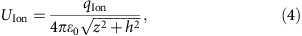


where *h* is the ion distance from the CNT centre axis, *z* is the distance along the CNT axis relative to the adsorption site and *q*_Ion_ is the charge of the ion. The band structure modified by *U*_Ion_ is shown in Fig. 7a, and it forms a potential barrier in the CNT valence band, reducing current, increasing the valence band-Fermi energy difference and shifting the threshold gate voltage towards more negative values. An additional result of this study is that the ion potential forms a single bound electron state near the conduction band edge. The state energy is too high to contribute to device electrical behaviour in the *p*-type ON state; however, the result is nonetheless interesting and may have important implications for electrical behaviour in *n*-type devices. The nanotube experimental *I*–*V*_G_ data with and without ion exposure were fit with model parameters (Supplementary Note 3), and the resulting curves plotted in Fig. 7b along with the experimental data. When tunnelling effects are taken into account (see Supplementary Note 4 and Supplementary Figs 3 and 4), the estimated value for *q*_Ion_ based on the fit was +0.18 elementary charges, which agrees with the DFT results showing significant charge transfer to the adsorbed ion. The value for *h* was found to be 7 Å, which also corresponds well with DFT results for the equilibrium N_2_^+^–CNT axis separation of 6 Å. The fit values for *q*_Ion_ and *h* are independent of the fit value for *α*_ion_, as the first two change the threshold voltage, while the later changes the subthreshold swing.

### Decreased gate efficiency

The defect potential gate efficiency *α*_ion_ was found to be 14%, which is ∼3.5 times lower than the gate efficiency for the device as a whole (50%). Similar changes in the subthreshold swing are often observed in silicon FET devices when mid-gap energy defect states are created at the Si/SiO_2_ interface. These traps add an effective capacitance term *C*_it_=*q*^2^*D*_it_ associated with the defect density of states into the calculation for subthreshold swing^32^. This mechanism is not sufficient, however, to explain the increase in the subthreshold swing observed in the experimental *I*–*V*_G_ data, as these devices are clean, as-grown, suspended CNTs, with very low defect concentrations. In addition, there is insufficient evidence from theoretical modelling of the ion–CNT system for a large enough density of mid-gap states to warrant this mechanism as the sole explanation for the observed increase in the subthreshold swing.

An alternative mechanism is proposed to explain the decrease in gate efficiency and increase in subthreshold swing. When the adsorbed ion modifies the potential along the length of the CNT as depicted in Fig. 7, there is an additional stray capacitance contribution from the nearby conducting portion of the CNT (*C*_CNT_), which is added to the total system capacitance *C*_Total,ion_=*C*_G_+*C*_S,D_+*C*_CNT_, as shown in Fig. 7c. When this additional source of stray capacitance becomes available, the gate efficiency is decreased, leading to an increased subthreshold swing, according to equation 2. This additional capacitance can also be thought of as capacitive coupling to the partially filled density of states in the valence band of nearby sections of the CNT. Owing to the ion potential, these states are mid-gap in the defect region, as can be seen in Fig. 7a. Hence, this coupling is analogous to the effective capacitance of interface traps in silicon FET devices. Given the gate capacitance of 8 pF m^−1^, this reduction in alpha corresponds to an additional stray capacitance of *C*_CNT_=40 pF m^−1^. For comparison, the quantum capacitance of an individual CNT is 368 pF m^−1^. These values are compatible with the proposed model of capacitive coupling to the partially filled density of states of nearby CNT; however, a more thorough, quantitative analysis of the electrostatics involved is needed for a complete understanding of the changes observed in the subthreshold swing. The Landauer transport model with ion potential included fits the data reasonably well, except for in the case of the OFF state, which is dominated by the noise floor and leakage currents, and above-threshold, where the measured current is lower than that predicted by the model. This discrepancy might be accounted for by defect potential-induced charge-carrier scattering.

## Discussion

The transient switching events observed during exposure to ionized gas are attributed to the ionized gas molecules from the surrounding atmosphere adsorbing on the CNT and behaving as defects that modify the electrical properties of the CNT channel. This hypothesis is well supported by the data in Figs 2 and 3, which show discrete quantized resistance increases in the CNT resistance when exposed to ionized gas, and also by the fact that the effect is only observed during exposure to positive ions.

It may seem surprising that a potential well caused by such a small charge could reduce the current passing through the CNT by over two orders of magnitude. On further inspection, however, this seems reasonable. The potential barrier created by the above ion configuration has a barrier height of ∼0.37 eV and a full-width half-maximum of 2.5 nm. This alone would pose a significant impediment to charge transport, but perhaps not to the degree seen in the data. This figure for barrier width, however, ignores the energy dependence of charge carrier transport. For the time series data, the gate bias voltage was held at 0 V, which corresponds approximately to the threshold voltage of this device. In this state, the Fermi energy is well inside the forbidden band gap, and thus only the charge carriers closest to the valence band edge contribute to charge transport. A weighted average of the barrier width based on the Fermi distribution of carrier population yields a much larger *effective* barrier width of 22 nm. Thus, when the device is biased into this subthreshold state, it is very sensitive to external potential variations. However, when the device is fully turned on (for large negative gate voltages), the potential perturbation has a much smaller effect, as is seen in Fig. 7. The extreme sensitivity to electrically charged defects observed here can also be understood by contrasting the CNT with two- and three-dimensional systems. In these systems, charge carriers can bypass localized potential barriers by going around them. This is not possible in the severely restricted current path of the 1D CNT.

In summary, we report on the extreme sensitivity of nano-electronic devices to single gaseous ion adsorption, which is attributed to single-ion-induced charge depletion in the 1D channel. Ion adsorption on the CNT is modelled with DFT, which predicts large binding energies, an ion–CNT surface separation of 3 Å and a significant charge transfer. The gate voltage dependence of the CNT current during ion exposure is modelled with a numerical solution of the discretized quantum mechanical Hamiltonian combined with a Landauer electrical transport model, with good agreement between theoretical modelling and experimental results. These experiments have profound impact on our current understanding of the interaction of single ions in 1D nano-electronic materials, and demonstrate the basis for a powerful system to study ion adsorption dynamics^33^ at the single-ion level, and also charge transport in CNTs as well as other nano-electronic systems such as single-atom transistors^34^, nanowires^35^ and quantum dots^36^. Equally important is the demonstration of a new single-ion/CNT-based 1D heterostructure, with novel electronic properties such as resonant tunnelling and single-electron bound states. In addition, the response of nano-electronic devices to ionized gasses has implications for microelectronic devices operating while being exposed to ionizing radiation, as, for instance, in the space radiation environment^37^,^38^. The effects observed here raise the prospect of sensitive gas detectors with short exposure times and miniscule detection limits operating at room temperature with almost complete noise immunity. Several important questions remain, such as the cause for the large differences observed in ion–surface adsorption lifetime, and also the nature of interactions between the CNT and ions of different chemical compositions.

## Methods

### CNT device fabrication

The isolated, suspended CNTs were grown by using chemical vapour deposition at temperatures between 800 and 900 °C, argon-bubbled through ethanol as the carbon source and lithographically defined catalyst islands consisting of a Fe–Mo mixture on an oxide support^27^. After chemical vapour deposition, nearly defect-free, isolated, suspended CNTs are produced, as evidenced by Raman spectroscopy. CNTs span 500-nm-deep trench structures with widths from 500 nm to 2 μm, formed in a silicon substrate with 1 μm SiO_2_ per 100-nm-low stress SiN_*x*_ (Fig. 1a).

### Electrical characterization and ion generation methods

Electrical measurements were performed with an Agilent 4,155 semiconductor parameter analyser with *V*_ds_=0.1 V, and all measurements were performed at room temperature. Gaseous ions were generated using ionizing radiation exposure with 50-MeV protons from the 88″ cyclotron facility at the Lawrence Berkeley National Laboratory, and also with gamma radiation (1.17/1.33 MeV) from a Co-60 gamma ray source at The Aerospace Corporation. Gaseous ions were also generated using high-voltage corona discharge from commercially available benchtop air ionizers. The resulting ions generated by these methods are in thermal equilibrium with the surrounding neutral gas molecules.

### Theoretical modelling and simulation

DFT calculations were performed using the plane-wave pseudopotential method implemented using the Quantum ESPRESSO DFT package (v. 5.1)^39^, using the spin-polarized Perdew-Burke-Ernzerhof (PBE) gradient-corrected functional^40^. Further details on DFT implementation and results are discussed in Supplementary Note 2. Device-level numerical simulations were performed with Matlab on an engineering workstation. Eigen functions of Schrödinger's equation were obtained by solving a discretized Hamiltonian matrix, and energy-dependent transmission coefficients through the ion potential barrier were calculated using the propagation matrix method^30^. The details on implementation of the Landauer transport model are reported elsewhere^28^,^31^.

## Additional information

**How to cite this article:** Bushmaker, A. W. *et al*. Single-ion adsorption and switching in carbon nanotubes. *Nat. Commun.* 7:10475 doi: 10.1038/ncomms10475 (2016).

## Supplementary Material

Supplementary Information Supplementary Figures 1-4, Supplementary Notes 1-4 and Supplementary References
