# Supplementary material for: Single-ion adsorption and switching in carbon nanotubes
Source: Nat Commun. 2016 Jan 25;7:10475. doi: 10.1038/ncomms10475 (PMC4737755; doi:10.1038/ncomms10475)
Supplement: Supplementary Information — Supplementary Figures 1-4, Supplementary Notes 1-4 and Supplementary References [file ncomms10475-s1.pdf]

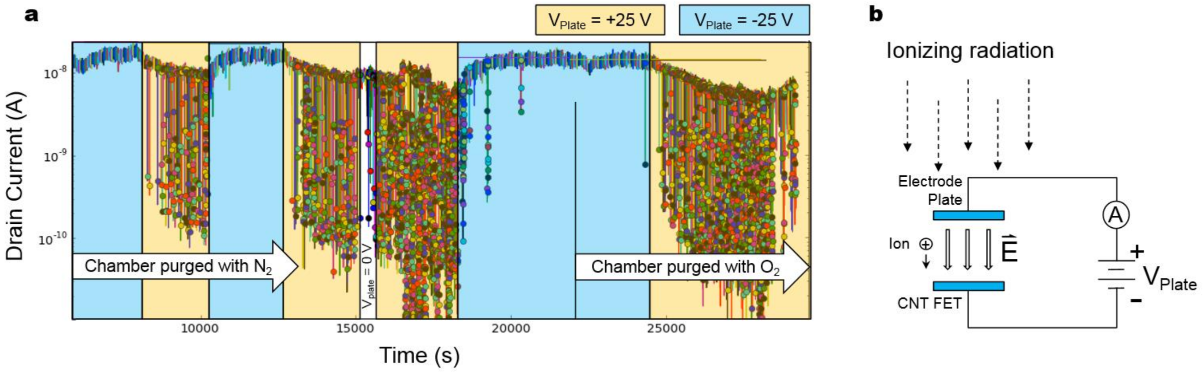

Supplementary Figure 1: Ion-polarity selective exposure. **a** Drain current plotted versus for a CNT FET exposed alternately to positive (yellow) and negative (blue) ions, and **b** ionization drift chamber cartoon diagram, illustrating the application of electric fields for ion polarity selection.

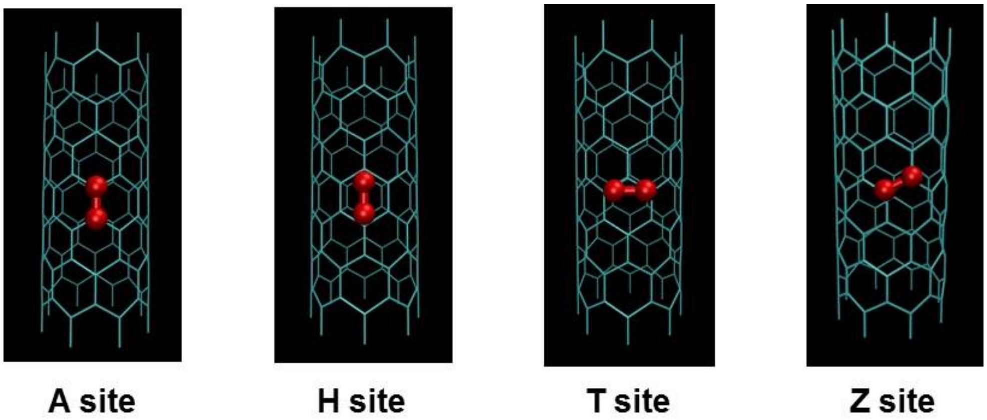

Supplementary Figure 2: Geometry of (8,0) single-walled CNT with N_2 adsorbate at the four adsorption sites investigated by this study.

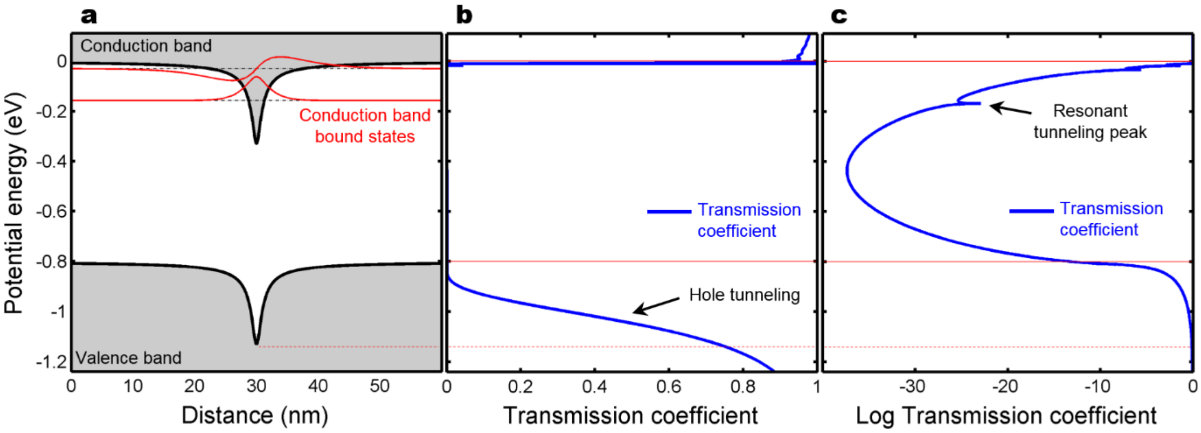

Supplementary Figure 3: Energy dependent transmission coefficient. **a** Ion potential well, with bound conduction band states, **b** calculated transmission coefficient showing finite tunneling through the ion potential barrier, and **c** log plot of the transmission coefficient, showing a small resonant tunneling peaks at the bound conduction band state energies.

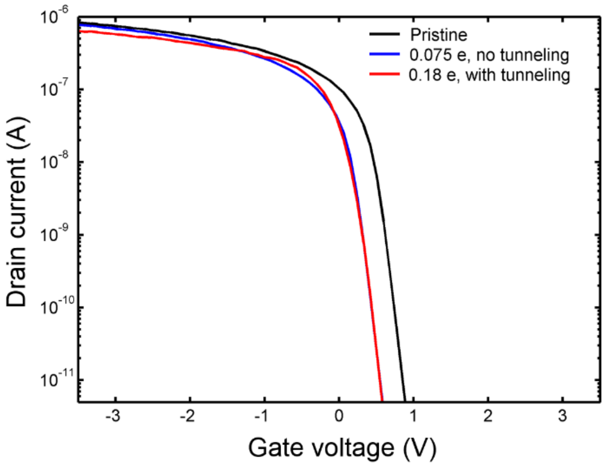

Supplementary Figure 4: Effect of tunneling on I-V_G curve. The primary effect of tunneling through the ion potential is nearly indistinguishable from reduced potential depth/ion charge.

Supplementary Note 1. Ion-Polarity Effects

In order to determine the effect of positive versus negative polarity ions on the electrical properties of the CNT, an ionization drift chamber was constructed to apply electric fields to the ionized gas inside the chamber. Supplementary Fig. 1a shows drain current versus time during exposure to ionized nitrogen and oxygen gas with alternating electric field polarity in the chamber. Over 5 million data points are plotted in Supplementary Fig. 1a, in which thousands of switching events were observed. The circular dots in the plot represent hits from an automated switching event detection algorithm. When the field polarity was such that positive ions were driven towards the CNT, the switching events were frequent; when negative ions and electrons were driven towards the CNT, the switching events mostly ceased occuring. At the start of the experiment, ionized nitrogen gas was used, which is well approximated as a mix of positive ions and free electrons. After a later time, the chamber was purged with oxygen, which has a high electron affinity, resulting in a substantial negative ion population from electron capture by neutral oxygen molecules[1]. Almost no switching events were observed during exposure to either free electrons or negative oxygen ions. The increases and decreases in nominal drain current after polarity switches are thought to be caused by surface charging of exposed Si_3N_4 dielectric around the CNT FET. The electric field in the ionization chamber was adjusted to a value slightly above the saturation field strength, where all ionized charge created was collected at the electrode plate, minimizing ion recombination losses. This field strength was, however, well below the threshold for impact avalanche multiplication.

Supplementary Note 2. DFT Binding Energy Calculations

DFT calculations were performed to investigate the binding energies of neutral N_2 and N_2^+ at 4 different surface sites (A, H, T and Z) based on earlier work by Dag et al. on the adsorption of O_2 on (8,0) single-walled CNTs[2] (See Supplementary Fig. 2). Ion cores are represented by ultra-soft pseudopotentials (C.pbe-rrkjus.UPF, N.pbe-rrkjus.UPF) available from www.quantum-espresso.org, and a plane-wave basis with kinetic energy cut-off of 280 eV are used to model the valence electrons. Test calculations of adsorbate binding energies performed with higher kinetic energy cut-off values showed that higher cut-off values did not significantly change the results. Semi-empirical van der Waals interactions were included in binding energy calculations[3,4]. DOS calculations was performed using the tetrahedra method[5].

The binding energies reported in the main body of the manuscript reflect the binding energies averaged over all four binding sites and the reported standard deviation reflects differences in binding energy at each adsorption site for both the neutral and ionized N_2 adsorbate. The relatively weak binding energies reported here for neutral N_2 adsorbate are consistent with the findings Dag et al. reported for neutral, ground state O_2.

Supplementary Note 3. Gate Voltage Dependence Fitting Parameters

The gate voltage dependence of the CNT while in the defected state (Fig. 7, main body) was repeatable, but due to the transient nature of the events it was necessary to plot several IV curves while the CNT was in the for the lower, degraded current state in order to cover the full gate voltage range. For the Landauer model calculation, the band gap was set to 0.8 eV[6,7], and the gate capacitance used was 8 pF/m, chosen based on curve fitting of the pristine IV curve, and also from previously determined values for these devices[8]. The electron mean free path, gate capacitance, α, and contact resistance were adjusted until the theoretical model fit the pristine data curve. The resulting values for high energy electron mean free path, contact resistance, and α were 2.5 µm, 23 kΩ, and 50%, respectively. These values are in line with those previously reported in the literature[7,9,10]. In order to fit the defected nanotube IV curves taken during exposure to ionized gas, the ion potential and reduced gate efficiency were adjusted until the resulting curve fit the experimentally measured data. Other than these two parameters, the fitting of the defected data used the same values for electrical properties as with the pristine CNT fit. The details on implementation of the Landauer transport model are reported elsewhere[9,11].

Supplementary Note 4. Tunneling Transmission Coefficient Calculations

Since the ion potential is relatively narrow, there is a substantial probability for carrier tunneling. In order to investigate this effect, energy dependent transmission coefficients through the ion potential barrier were calculated using the propagation matrix method[12] with a hyperbolic band dispersion[13], and the results of these calculations are shown in Supplementary Figs. 3 and 4. Supplementary Fig. 3a shows the ion potential with an ion charge q_{Ion} of 0.18 elementary charges, and Supplementary Fig. 3b shows the transmission coefficient calculated for the potential in Supplementary Fig. 3a, confirming that there is indeed substantial hole tunneling through the ion potential. Supplementary Fig. 3c shows the log of the transmission coefficient plotted versus energy, and shows small resonant tunneling peaks at the bound conduction band state energies. Supplementary Fig. 4 shows the I-V_G curves calculated with the Landauer transport model using the transmission coefficient for the ion potential with and without tunneling, and gives insight into the effect of tunneling on the shape of the I-V_G. The curves plotted in Supplementary Fig. 4 were calculated for transmission through the ion potential only, without regard for the behavior of the entire device as a whole. There is significant hole tunneling through the ion potential; however, the primary effect on the shape of the I-V_G curve is simply to reduce the threshold voltage shift, which is nearly indistinguishable from a reduced adsorbed ion charge q_{Ion}, as shown in Supplementary Fig. 4. The effective ion charge q_{Ion} required to fit the experimental data in the main body using the device level model without tunneling was +0.075 elementary charges, and factoring in tunneling gives an actual ion charge of q_{Ion} = +0.18 elementary charges.

Supplementary references:

1. Knoll, G. F. *Radiation detection and measurement*. 4th edn, (John Wiley & Sons, 2010).
2. Durgun, E. *et al.* Systematic study of adsorption of single atoms on a carbon nanotube. *Phys. Rev. B* **67**, 201401 (2003).
3. Grimme, S. Semiempirical GGA-type density functional constructed with a long-range dispersion correction. *Journal of Computational Chemistry* **27**, 1787-1799, doi:10.1002/jcc.20495 (2006).
4. Barone, V. *et al.* Role and effective treatment of dispersive forces in materials: Polyethylene and graphite crystals as test cases. *Journal of Computational Chemistry* **30**, 934-939, doi:10.1002/jcc.21112 (2009).
5. Blöchl, P. E., Jepsen, O. & Andersen, O. K. Improved tetrahedron method for Brillouin-zone integrations. *Phys. Rev. B* **49**, 16223 (1994).
6. Weisman, R. B. & Bachilo, S. M. Dependence of optical transition energies on structure for single-walled carbon nanotubes in aqueous suspension: an empirical Kataura plot. *Nano Lett.* **3**, 1235-1238 (2003).
7. Bushmaker, A. W., Deshpande, V. V., Hsieh, S., Bockrath, M. & Cronin, S. B. Direct observation of the Born-Oppenheimer approximation breakdown in metallic carbon nanotubes. *Nano Lett.* **9**, 607-611 (2009).
8. Amer, M. R., Bushmaker, A. & Cronin, S. B. The influence of substrate in determining the band gap of metallic carbon nanotubes. *Nano Lett.* **12**, 4843-4847 (2012).
9. Bushmaker, A. W., Amer, M. R. & Cronin, S. B. Electrical Transport and Channel Length Modulation in Semiconducting Carbon Nanotube Field Effect Transistors. *IEEE Trans. Nanotechnol.* **13**, 176-181, doi:10.1109/tnano.2013.2289386 (2014).
10. Pop, E. *et al.* Negative differential conductance and hot phonons in suspended nanotube molecular wires. *Phys. Rev. Lett.* **95**, 155505-155508 (2005).
11. Rahman, A., Jing, G., Datta, S. & Lundstrom, M. S. Theory of ballistic nanotransistors. *IEEE Trans. Electron Devices,* **50**, 1853-1864 (2003).
12. Levi, A. F. J. *Applied quantum mechanics*. (Cambridge University Press, 2006).
13. Léonard, F. *The Physics of Carbon Nanotube Devices*. (William Andrew Inc., 2008).
